# The potential impact of an anti-stigma intervention on mental health help-seeking attitudes among university students

**DOI:** 10.1186/s12888-020-02960-y

**Published:** 2020-11-25

**Authors:** Shazana Shahwan, Jue Hua Lau, Chong Min Janrius Goh, Wei Jie Ong, Gregory Tee Hng Tan, Kian Woon Kwok, Ellaisha Samari, Ying Ying Lee, Wen Lin Teh, Vanessa Seet, Sherilyn Chang, Siow Ann Chong, Mythily Subramaniam

**Affiliations:** 1 Research Division, Institute of Mental Health, Buangkok Green Medical Park, 10 Buangkok View, Singapore, 539747 Singapore; 2 School of Social Sciences, Nanyang Technological University, 50 Nanyang Avenue, Singapore, 639798 Singapore

**Keywords:** help-seeking, mental illness, stigma, intervention

## Abstract

**Background:**

The reluctance of young adults to seek mental health treatment has been attributed to poor mental health literacy, stigma, preference for self-reliance and concerns about confidentiality. The purpose of this study was to examine the potential impact of an anti-stigma intervention that includes education about depression, information about help-seeking as well as contact with a person with lived experience, on help seeking attitudes.

**Methods:**

A pre-post study design was employed. Changes in help-seeking attitudes were measured using the Inventory of Attitudes towards Seeking Mental Health Services (IASMHS) immediately post-intervention and after 3 months. Sociodemographic data, information on past experiences in the mental health field and contact with people with mental illness were collated. Three hundred ninety university students enrolled in the study. Linear mixed models were used to examine the effects of the intervention.

**Results:**

Scores on all subscales of the IASMHS, Psychological Openness (PO), Help-seeking Propensity (HP) and Indifference to Stigma improved significantly post-intervention and at 3-month follow-up compared to pre-intervention, with HP demonstrating the highest effect size. However, a significant decline was observed on all three scales at 3-month follow-up compared to post-intervention. Gender, having friends/family with mental illness, and previous experience in the mental health field moderated the intervention effects for the PO and HP subscales.

**Conclusion:**

The study showed that the brief anti-stigma intervention was associated with improvements in help-seeking attitudes among university students with differential effects among certain sub-groups. As the beneficial outcomes appeared to decrease over time, booster sessions or opportunities to participate in mental health-related activities post-intervention may be required to maintain the desired changes in help-seeking attitudes.

The reluctance of young people to seek professional help for mental health problems poses a challenge to effective early intervention approaches [1, 2]. The Theory of Planned Behaviour (TPB) has been used as a framework for understanding the factors that affect decisions to seek professional help [3, 4]. TPB posits that three cognitive components, namely *attitude, subjective norm* and *perceived behavioural control,* are important in explaining behavioural intention and subsequently behaviour. People are more likely to engage in a health-related behaviour if they perceive the usefulness in engaging in the behaviour (attitude), if they feel that people whose views they value think they should carry out the behaviour (subjective norm), and if they have the necessary resources and opportunities to engage in the behaviour (behavioural control). An individual’s appraisal of his or her likelihood of performing a given behaviour is often used as a proxy for his or her intentions to engage in that behaviour. Background factors such as age, gender, education and cultural factors have been found to influence these cognitive components [4].

Systematic reviews identifying barriers and facilitators of mental health help-seeking in young people have revealed several key factors. Stigma and embarrassment about seeking help emerged as the most prominent barrier in Gulliver et al’s study [5], while disclosure and confidentiality concerns topped treatment-seeking barriers in Clement et al’s study [6]. Poor mental health literacy or the lack of knowledge regarding recognition, prevention and treatment of mental illnesses was another major barrier highlighted in other studies [5, 7, 8]. There is accumulating evidence to show that better mental health literacy is associated with positive attitudes towards seeking professional help and using mental health services [9]. A preference for self-reliance and low perceived need for care was listed as another salient barrier to mental health help-seeking [5, 6, 10]. For males, in particular, gender stereotypes (masculinity, stoicism, restricted emotionality) may interact with mental illness stereotypes (character weakness) to intensify the effect of stigma on help-seeking [11]. Positive past experiences with mental health services and social support on the other hand were facilitators to help-seeking. Social support facilitates help-seeking through the identification of appropriate sources of help and encouragement by confidants which may reduce the stigma of help-seeking [12]. Together, these studies suggest that appropriate service use among students will increase through interventions to change students’ attitudes, knowledge and beliefs about mental health.

Consistent with TPB, much intervention research on help-seeking has focused on improving help-seeking attitudes and intentions by targeting the main barriers to treatment. These interventions include [13]: *educational interventions* (to increase mental health literacy), *de-stigmatisation* (which is aimed at reducing the stigma surrounding mental disorders) often involving *contact with a person with lived experience*, and *providing help-seeking source information* (where to find potential providers of help) [13] that can be delivered either as a standalone or in combination.

A meta-analysis of 98 studies ranging in terms of quality, population, settings and type and mode of interventions to improve mental health help-seeking reported that interventions with literacy or de-stigmatisation strategies led to short-term improvements in help-seeking outcomes [14]. Gulliver and colleagues’ selective systematic review of six randomised controlled trials examining help-seeking interventions for mental health targeted mainly at young adults showed that almost all interventions that delivered mental health literacy were likewise, associated with improved attitudes or beliefs about professional help-seeking at post-test [13]. Results however, were mixed for interventions delivering de-stigmatisation information, where only two out of three studies reported significant improvement in attitudes. Providing information about help-seeking sources were common to three interventions and all three successfully improved attitudes. This review, however, did not find discernible effects for contact with mental health service users. The latter finding was interesting as Mehta et al., in their review of 72 interventions aimed at reducing mental health related stigma, found that social contact appeared to be the strongest evidence-based type of intervention to reduce stigma [15]. This finding was also observed in Janoushka et al’s review of 23 studies examining the effectiveness of video interventions to destigmatise mental illness, where contact-based videos had greater impact on stigma than educational videos, with the effect lasting after 1 week [16].

Although the direct relationship between stigma and help-seeking has been found to be only small-moderately sized [6], Schomerus and Angermeyer maintain that it is important to see stigma as part of a larger network of beliefs and other constraints deterring help seeking [17]. Clement and colleagues described the complex relationship in a detailed conceptual model [6] that includes the hypothesis that stigma may deter help-seeking through two routes: 1) by people wanting to avoid the label that receiving formal care often brings, so as to escape public stigma, and 2) by the desire to avoid experiencing internalized stigma such as shame and embarrassment [18]. Additionally, Clement and colleagues’ conceptual model captures factors that are not considered to be stigma-related such as wanting to handle the problem on one’s own and low perceived need for care, but may affect help-seeking when an individual holds stigmatising views about themselves [6].

Most of the evidence for the interventions described above were limited to immediate to short-term outcomes. Few studies have examined outcomes in the longer term. Two such studies were conducted in Tokyo, Japan among college students: Koike and colleagues [19] and Yamaguchi and colleagues [20] compared the effects of filmed social contact (FSC) and self-instructional Internet search (INS) interventions repeated at 2-month intervals on mental health literacy and stigma outcomes with that of controls at mid and long term. The effects of both these interventions on the two outcome measures peaked at 1 month after the initial intervention [20]. However, at 12-month follow-up, the FSC group was more successful at reducing stigma compared to the INS and no intervention control group [19]. At 24 months, the FSC group showed significant improvements in the behavioural intentions (for social contact with people with mental illness) compared to the control but not the INS group [20]. These studies did not examine the impact of the interventions on help-seeking.

The Singapore Mental Health Study (SMHS) 2016 was a nationwide study, where one of the aims was to investigate the treatment gap for mental disorders in Singapore. The SMHS 2016 showed that among the population who had the highest treatment gap were those with tertiary level education [21]. Individuals in tertiary institutions have a special significance as they represent a large number of people who are at an age when mental disorders first present (onset) and may have lasting implications if not treated properly [22]. In Singapore, meritocracy is highly regarded and is embodied in its highly competitive education system [23]. Thus, those pursuing tertiary education are most likely to face pressures to be high-achieving and resilient. They may fear being perceived otherwise once assigned a diagnosis and prefer to deal with the problem on their own [21].

We therefore developed an intervention to reduce stigma and improve help-seeking attitudes for this target group. The intervention was informed by earlier literature and comprised an educational intervention delivered in combination with sharing by a person with lived experience. The intervention focused on major depression due to the high prevalence of the disorder in young people in Singapore as found in the nationwide study [24].

Although earlier studies have shown that more intensive interventions produce better outcomes, extended and/or repeated interventions may not always be feasible. We instead focused on intervention components that have been found to be most effective to maximise outcomes. The first aim of the current study was to examine the impact of our stigma-reduction intervention on attitudes towards help-seeking, as few studies have examined it. A university setting offers an opportune venue for examining cognitive factors influencing help seeking. This is because most universities offer free onsite counselling services, thus reducing the likelihood of structural barriers such as cost and availability. Secondly, we were interested to find out the extent to which brief interventions such as ours could have a longer-term impact. Building on Koike and colleagues’ work [19] which showed that positive outcomes peaked at 1 month, we extended the follow-up to a 3-month period. We hypothesized that help-seeking attitudes will improve post-intervention and the benefits will be retained at 3 months’ follow up.

A secondary aim of the study was to examine the correlates and explore the moderators of help-seeking attitudes. We also hypothesized that those who had social contacts with mental illness and experience in mental health would have better attitudes towards help-seeking by virtue of their deeper understanding of mental health compared to those who do not [15, 25]. To the best of our knowledge no known study examining the impact of an anti-stigma intervention on help-seeking attitudes has been done in Singapore. This study will inform the potential impact of a combined education and contact-based intervention in our local setting and populations with similar cultural composition.

## Methods

### Sample

Data was collected as part of the Advancing Research Towards Eliminating Mental Illness Stigma (ARTEMIS) study, an interventional research project targeted at students from one of the 6 local publicly-funded autonomous universities, which is aimed at increasing knowledge about depression and improving attitudes towards mental illness and seeking professional help. The associations between this intervention and mental health literacy and stigma outcomes have been published [26, 27]. A total of 390 university students from the local university were recruited for the study from October 2018 to April 2019. The inclusion criteria for participation were students aged between 18 and 35 years, willingness to provide e-mail address for recontact at 3-month follow-up and literacy in English. The exclusion criterion was not being a student of the university. Research assistants checked that the email addresses of students who enrolled were not repeated and the same group of researchers who received the students on the day of the intervention were present at all 9 session to avoid double compilation. This study was reviewed by the National Healthcare Group, Domain Specific Review Board (DSRB Reference number: 2018/00695).

### Procedure

The team worked together with designated university staff to attain the target sample size. The researchers were not part of the university and did not make initial contact with students. The researchers' contact was limited only to students who enrolled in the study. The university staff employed various strategies for recruitment. Mass e-mail was sent out by the university staff to various faculties in the university (e.g. Arts, Humanities, Engineering, Business, Sciences) and/or halls of residences (i.e. on-campus housing for students), inviting students to indicate their willingness to participate in the study through an online event management tool managed by the researchers. The study was also advertised by putting up a post on the university’s Facebook groups with the sign-up page link created by the event management tool, and by putting up posters with a QR code leading to the same sign-up page for those interested in participating. Those who indicated their willingness were sent consent forms by e-mail. Specific instructions were additionally given to students under 21 years – the age of majority in Singapore – to bring along their consent form with written parental consent. On the day of the session, each participant’s informed consent was confirmed by a research staff, and all were asked to clarify any doubts related to the study or study procedures. Recruitment ceased when the recruitment target was attained and statistical analysis showed that the study was sufficiently powered. As the total number of students reached was not captured (e.g. students’ emails were masked in the mass email blasts, use of several recruitment strategies), the response rate cannot be determined.

### Design

A pre-post study design without a control group was adopted for the study. Pre-intervention data was collected on the same day immediately before the intervention using a set of paper and pencil questionnaires described below, and post-intervention data was collected immediately after the intervention using an identical set of questionnaires. A research assistant collected the pre/post questionnaires and counted the sets of questionnaires to ensure that the number of sets tallied with the number of students present at the venue. 3-month follow-up data using the same measures was collected via an online form that was accessed by students using a URL unique to each participant that was emailed to them. This URL could not be used once the online form was completed by the participant to avoid misuse of the survey link. A mass reminder email was sent 2 weeks after the follow-up email was sent. A total window period of 4 weeks was given to complete the survey from the initial follow-up contact before the survey link was deactivated. The pre-post and 3-month follow-up questionnaire each took about 20 min to complete.

### Intervention

All participants underwent the same intervention which consisted of three main sections: 1a) a lecture delivered by a mental health professional providing information about depression including prevalence, biopsychosocial causes, treatment options and its efficacy, local avenues for seeking help, and helpful and unhelpful responses to a friend experiencing depression symptoms; b) a video by the WHO entitled ‘I had a black dog, his name was depression’, narrated by a male voice of the experience and recovery from depression (https://www.youtube.com/watch?v=XiCrniLQGYc) and inserted prior to providing the information on local avenues for seeking help; 2) a candid sharing by a person with lived experience about her reflections on her episodes of illness and recovery journey; and 3) a question-and-answer session with a consultant psychiatrist (CSA) as well as a senior mental health researcher (MS). The intervention lasted approximately 50 min. Participant interaction was encouraged, with the presenter asking a few questions (e.g. why do you think men seek help less frequently than women?), inviting responses from participants before providing the prepared responses. Table 1 presents aspects of the intervention that has the potential to influence the attitude, subjective norm, and perceived behavioural control components of the TPB towards mental health help-seeking.

**Table 1:** Components of TPB incorporated into the ARTEMIS intervention

TPB component targeted	Definition	Identified barrier targeted	ARTEMIS strategy
**Attitude (towards seeking mental health services)**	Extent to which an individual has a positive or negative appraisal toward mental health help-seeking	Treatment fears Expectation that treatment is not helpful	(L) Biopsychosocial etiological model of mental illness. (L) Efficacy of medical, psychological and combined treatment (L) Treatment gap and consequences of delayed treatment seeking (Q&A) Psychiatrist addressing concerns about medication side effects (SC/V) Role of treatment in recovery journey by social contact and narration in Black Dog video
**Subjective Norm**	Perceived social pressure/approval to seek mental health treatment	Seeking help for mental illness is frowned upon	(L) High prevalence of depression among tertiary students (L, V, Q&A) Information that help-seeking improves life outcomes (SC) Role modelling by social contact
**Perceived Behavioural Control**	Extent to which a person perceives mental health help-seeking as easy or difficult.	Do not know: When to seek help, Where and How to seek help Concerns about affordability Concerns about confidentiality	(L) Signs and symptoms of depression (L, SC, Q&A) Avenues for help-seeking (Q&A) Psychiatrist responses to confidentiality of treatment

*TPB* theory of planned behaviour, *L* lecture, *SC* social contact, *V* video, *Q&A* question and answer with psychiatrist

Each session was restricted to 50–80 students depending on the size of the venue. The sessions were sub-divided by residence in halls and/or faculties depending on the groups that the mass email was sent out to, with the venue being localised for the participants’ convenience. The last 2 sessions comprised a mix of faculties and held in a central venue of the university as the last 2 email blasts were repeated with a cluster of faculties to so as to attain a sufficient number of students from each faculty who wished to participate but had missed earlier sessions and to attain sufficient group sizes so as to close recruitment in the shortest possible time. This was done to minimise disruption to the university’s schedule, burden on staff and venue usage. All enrolled participants who met the inclusion criteria were accepted; no quota was placed in terms of age and gender for each session. The same material and format were used in all 9 sessions, 6 of which were conducted by the same person.

### Measures

The following sociodemographic information were collected: Age, gender and ethnicity. Due to the small number of participants from ethnic minority groups, Malay, Indian and other ethnic groups formed a separate category for the purpose of analysis. Additionally, to explore if social contact and prior knowledge of mental health was associated with help-seeking attitudes, the following questions were asked: “Do you have a close friend or family member who has a mental illness” (Yes/No), “Do you have past experiences within the mental health field” (Yes/No).

The Inventory of Attitudes toward Seeking Mental Health Services (IASMHS) is a 24-item scale designed to measure an individual’s attitudes towards seeking mental health services, which comprises 3 factors: Psychological Openness, Help-seeking Propensity, and Indifference to Stigma [28]. Each item was rated on a scale of 0–4 (Disagree-Agree). Subscale scores are the sum of scores for each item on that scale. *Psychological Openness* reflects the degree to which an individual is open to acknowledging the presence of a psychological problem, and seeking professional care for such a problem. *Help-seeking Propensity* reflects one’s willingness and perceived ability to seek help for psychological problems. *Indifference to Stigma* refers to how concerned an individual would feel if significant others were to discover that they were receiving psychological care. Ajzen’s 3 components of attitude, behavioural control and subjective norms correspond to these 3 subscales respectively [29]. The IASMHS was selected as it was designed to be a multifactorial, theoretically-based attitude inventory grounded in the TPB. The three-factor model of the IASMHS was validated in two studies which employed different samples [30, 31] but has not been validated in Singapore. The internal consistency of the Psychological Openness, Help-seeking Propensity, and Indifference to Stigma subscales at pre-intervention were moderate to high, with Cronbach α values of 0.67, 0.70, and 0.81 respectively, comparable to that found among college students in Taiwan [32].

All measures were presented in standard English, which is officially the main language of instruction in Singapore’s education system.

### Statistical analysis

All statistical analyses were conducted using SPSS version 22. Frequencies and percentages were calculated for categorical variables, while means and standard deviations were calculated for continuous variables. Three linear mixed models were utilized to examine the effect of the intervention to account for missing data, individual heterogeneity, and longitudinal measures within the same individual. The ‘time’ variable (0 = pre-intervention, 1 = post-intervention, 2 = 3 months later) was included in each linear mixed model as both a random and fixed effect to adjust for the overall and individual variations in each IASMHS subscale (Psychological openness, Help-seeking propensity, and Indifference to stigma) score over time. Linear and quadratic effects, along with interaction terms and covariates were tested as fixed parameters. Each model was first constructed unconditionally (i.e. without any covariates) in order to examine the IASMHS subscale scores at the three time-points (pre, post, and 3 months), after which the following were entered as time-invariant covariates (i.e. covariate that do not change with time): i) sociodemographic factors (i.e. age, gender, ethnicity), ii) whether they had close friends or family with a mental illness, iii) whether they had past experience in the mental health field. Subsequently, the interactions between the linear and quadratic effects and each covariate were explored in order to account for any potential effect these interactions might have on the rate of change in IASMHS scores over time. Tested interaction effects that were not significant and did not significantly improve model fit based on -2LogLikelihood, Akaike Information Criterion, or Bayesian Information Criterion values were not included in the final model. Effect sizes comparing the pre-intervention scores to post-intervention and 3-month follow-up were calculated using the following formula using simple means and standard deviations: $Cohe{n}^{\prime }s\ d=\frac{\left( MeanTime2- MeanTime1\right)}{Pooled\ S.D.}$. With regard to effect size, previous literature has suggested that a Cohen’s d value of 0.2 to 0.5 represents a small effect, a value of 0.5 to 0.8 represents a medium effect, while a value of 0.8 or higher represents a large effect. Statistical significance for all analyses was set at the conventional alpha level of *p* <  0.05, using two-tailed tests.

## Results

### Characteristics of the sample

The characteristics of the sample at pre-post intervention and at 3-month follow-up are presented in Table 2. The sample consisted of 390 students from a university in Singapore. The majority of the sample were female (*n* = 235, 60.3%) and Chinese (*n* = 323, 82.8%). 166 (42.6%) participants had close friends or family with a mental illness, while 86 (22.1%) reported that they had past experience within the mental health field.

**Table 2:** Sociodemographic characteristics of the sample

	Pre- and Post-intervention (*n* = 390)		3-month follow-up (*n* = 324)	
	n	%	n	%
Gender				
Female	235	60.3	197	60.8
Male	155	39.7	127	39.2
Ethnicity				
Chinese	323	82.8	272	84.0
Others	67	17.2	52	16.0
Family or friends with mental illness^a^				
Yes	166	42.6	134	41.4
No	224	57.4	190	58.6
Past experience in mental health field^ab^				
Yes	86	22.1	75	23.1
No	301	77.2	247	76.2
	Mean	S.D.	Mean	S.D.
Age (in years)	22.28	2.26	22.25	2.24

^a^reported at pre-intervention

^b^Percentages do not add up to 100% due to missing data

### Psychological Openness scores over time

**Fig. 1:**
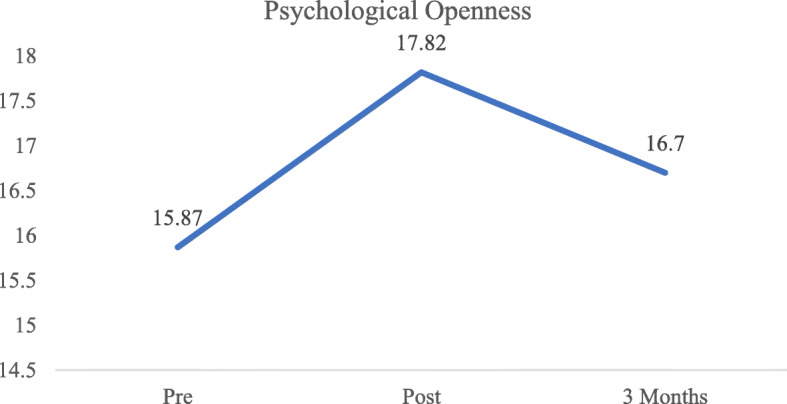
Mean Psychological Openness scores across time

As indicated in Table 3, the effect size of the intervention was small at both post-intervention (*d* = 0.37) and 3-month follow-up (*d* = 0.16). When compared to pre-intervention scores, scores at both post-intervention (*p* <  0.001) and the 3-month follow-up (*p* = 0.007) significantly increased. In contrast, compared to scores at post-intervention, there was a significant decrease (*p* <  0.001) at the 3-month follow-up. After adjusting for the effect of covariates, the results of the linear mixed model displayed in Table 4 indicate significant linear (B (unstandardized coefficient) = 4.05, 95% CI: 3.19–4.90, *p* <  0.001) and quadratic (B = − 1.57, 95% CI: − 1.96 - -1.19, *p* <  0.001) effects, representing an increase in Psychological Openness scores at post-intervention, but a decrease in scores at the 3-month follow-up (Fig. 1).

**Table 3:** Psychological Openness, Help-seeking Propensity, and Indifference to Stigma scores at pre-intervention, post-intervention, and at 3-month follow up

	Pre-intervention			Post-intervention			3-month			Effect size (Cohen’s *d*^a^)		*p* values^b^		
	n	mean	S.D.	n	mean	S.D.	n	mean	S.D.	Post-intervention	3-month follow-up	Pre vs Post	Pre vs 3-month	Post vs 3- month
Psychological Openness	388	15.87	5.17	385	17.82	5.38	324	16.71	5.57	0.37	0.16	**< 0.001**	**0.007**	**< 0.001**
Help Seeking Propensity	389	20.81	4.78	389	24.59	3.99	324	23.13	4.40	0.86	0.51	**< 0.001**	**< 0.001**	**< 0.001**
Indifference to Stigma	386	16.65	6.52	388	20.08	6.71	324	18.98	7.00	0.52	0.34	**< 0.001**	**< 0.001**	**0.002**

^a^ $$ Cohe{n}^{\prime }s\ d=\frac{\left( MeanTime2- MeanTime\right)}{Pooled\ S.D} $$

^b^ pairwise comparison of respective scores in unconditional linear mixed models, bold values denotes statistically significant values

**Table 4:** Estimates of Linear Mixed Models examining the effect of the intervention on the Inventory of Attitudes toward Seeking Mental Health Services

	Psychological Openness			Help-seeking Propensity			Indifference to Stigma		
	B	95% CI	*p*	B	95% CI	*p*	B	95% CI	*p*
Intercept	**6.82**	1.70–11.93	**0.01**	**14.17**	9.98–18.35	**< 0.001**	**10.21**	3.33–17.08	**0.004**
Time	**4.05**	3.19–4.90	**< 0.001**	**6.82**	6.07–7.58	**< 0.001**	**5.62**	4.71–6.52	**< 0.001**
Time^2^	**−1.57**	−1.96 – −1.19	**< 0.001**	**−2.83**	−3.19 – −2.47	**< 0.001**	**−2.21**	−2.65 – −1.77	**< 0.001**
Age (Years)	**0.26**	0.05–0.47	**0.02**	**0.31**	0.14–0.48	**< 0.001**	0.27	−0.01 – 0.55	0.06
Gender									
Female	**2.18**	1.14–3.23	**< 0.001**	−0.30	−1.11 – 0.51	0.47	1.31	−0.02 – 2.65	0.05
Male	ref			ref			ref		
Ethnicity									
Chinese	0.50	−0.67 – 1.67	0.40	−0.62	−1.57 – 0.34	0.21	−0.98	−2.55 – 0.59	0.22
Others	ref			ref			ref		
Family or friends with mental illness									
Yes	**2.59**	1.62–3.56	**< 0.001**	0.01	−0.73 – 0.75	0.98	0.88	−0.34 – 2.09	0.16
No	ref			ref			ref		
Past experience in mental health field									
Yes	**1.92**	0.84–2.99	**< 0.001**	**1.85**	0.75–2.94	**0.01**	0.43	−1.01 – 1.87	0.56
No	ref			ref			ref		
Interaction terms									
Time * Gender	**−0.56**	−1.08 – −0.03	**0.04**						
Time*Family or friends with mental illness	**−0.55**	−1.07 – − 0.03	**0.04**						
Time *Past experience in mental health field				**−1.82**	−3.41 – −0.23	**0.03**			
Time^2^ *Past experience in mental health field				**0.77**	0.01–1.52	**0.047**			

B – unstandardized regression coefficient; 95% CI: 95% confidence interval of B

Bold print denotes statistically significant B value

Results also indicated significant sociodemographic variables (Table 4), with females (B = 2.18, 95% CI: 1.14–3.23, *p* <  0.001) having higher psychological openness scores than males. Age was also positively associated with psychological openness scores (B = 0.26, 95% CI: 0.05–0.47, *p* = 0.02). Both having family or friends with mental illness (B = 2.59, 95% CI: 1.62–3.56, *p* <  0.001) and past experience in the mental health field (B = 1.92, 95% CI: 0.84–2.99, *p* <  0.001) were significantly associated with higher psychological openness scores.

**Fig. 2:**
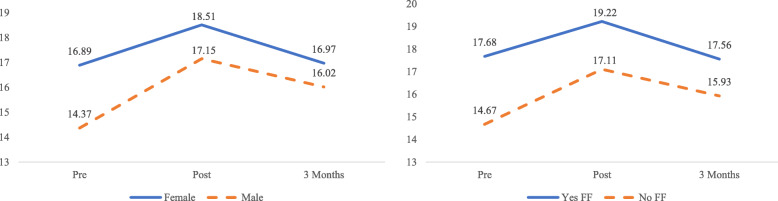
Left - Effect of intervention on Psychological Openness scores in Females and Males. Right- Effect of intervention on Psychological Openness scores among participants having family/friends with mental illness (Yes FF) and those who do not (No FF)

When interaction terms were added into the model, significant interactions were found between the linear effect and gender (B = − 0.56, 95% CI: − 1.08 – 0.03, *p* = 0.04), as well as having family or friends with a mental illness (B = − 0.55, 95% CI: − 1.07 – − 0.03, *p* = 0.04). Females had a smaller increase than males in psychological openness scores at post-intervention compared to baseline. Similarly, there was a smaller decrease in psychological openness scores in individuals without any friends or family suffering from mental illness than their counterparts (Fig. 2).

### Help-seeking Propensity scores over time

**Fig. 3:**
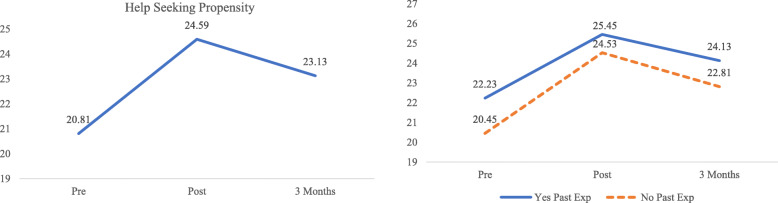
Left - Mean Help-seeking Propensity scores across time. Right – Effect of the intervention on Help-seeking Propensity scores among participants having past experience in a mental health (Yes Past Exp) and those who do not (No Past Exp)

The effect size of the intervention on help-seeking propensity scores was large at post-intervention (*d* = 0.86), and medium at the 3-month follow-up (*d* = 0.51). Compared to scores at pre-intervention, help-seeking propensity scores at both post-intervention (*p* <  0.001) and 3-month follow-up (*p* <  0.001) significantly increased. However, there was a significant decrease in scores at the 3-month follow-up as compared to post-intervention (*p* <  0.001) (Fig. 3). After adjusting for the effects of all covariates, results displayed in Table 4 indicate significant linear (B = 6.82, 95% CI: 6.07–7.58, *p* <  0.001) and quadratic (B = − 2.83, 95% CI: − 3.19 – − 2.47, *p* <  0.001) effects, denoting a significant increase in scores at post-intervention, but decrease at 3-month follow-up.

Of all the sociodemographic variables, only age and having past experience in a mental health setting were significantly associated with help-seeking propensity scores. Older students were more likely to have higher help-seeking propensity scores (B = 0.31, 95% CI: 0.14–0.48, *p* <  0.001), while those with past experience in a mental health setting were more likely to have higher scores than their counterparts (B = 1.85, 95% CI: 0.75–2.94, *p* = 0.01).

After testing interaction terms in the model, significant interactions were found between the linear and quadratic effects and having past experience in a mental health setting. Students with past experience in a mental health field tended to have a smaller increase in help-seeking propensity scores at post-intervention (B = − 1.82, 95% CI: − 3.41 – 0.23, *p* = 0.03), and had a smaller decrease in scores over the 3-month follow-up (B = 0.77, 95% CI: 0.01–1.52, *p* = 0.047) as compared to those without past experience (Fig. 3).

### Indifference to Stigma scores over time

**Fig. 4:**
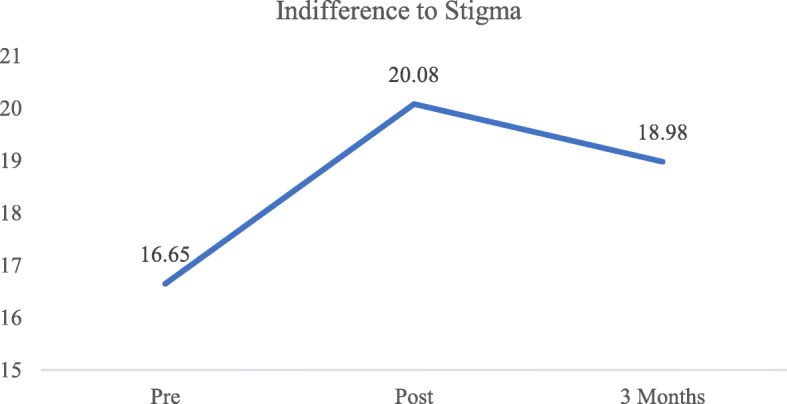
Mean Indifference to Stigma scores across time

As displayed in Table 3, the effect size of the intervention on indifference to stigma scores was moderate at post-intervention (*d =* 0.52) and small at the 3-month follow-up (*d* = 0.34). Scores at both post-intervention (*p* <  0.001) and the 3-month follow-up (*p* <  0.001) significantly increased when compared to scores at pre-intervention. However, when compared to scores at post-intervention, there was a significant decrease (*p* = 0.002) at the 3-month follow-up (Fig. 4). After adjusting for the effects of sociodemographic covariates, results displayed in Table 4 indicate both significant linear (B = 5.62, 95% CI: 4.71–6.52, *p* <  0.001) and quadratic (B = − 2.21, 95% CI: − 2.65 – − 1.77, *p* <  0.001) effects. There were no significant sociodemographic variables associated with indifference to stigma scores. None of the interaction effects tested were significant and did not significantly improve model fit (−2LL, AIC, BIC), and therefore not included in the final model.

## Discussion

The hypothesis that the anti-stigma intervention will result in improvements in attitudes towards help-seeking immediately post-intervention was supported with scores improving significantly from pre-test to post-test on all three subscales (Psychological Openness, Help-seeking Propensity, and Indifference to Stigma). This suggests that the intervention was successful in modifying all 3 cognitive components, which according to the TPB, influences behavioural intention.

The anti-stigma intervention was brief, lasting 50 min, and packed content that was associated with improvements in attitudes towards mental illness and help-seeking in other studies [13, 15, 33]. The intervention was tailored for the target group (e.g. discussion on prevalence of depression for their age group, using examples of causes and symptoms of depression presented in the context of a university student). Presentation of the biopsychosocial etiology of depression may have contributed to improved help-seeking attitudes by shifting causal attributions of the illness [16]. Our earlier analysis showed that the tendency to attribute depression to an individual’s personality was associated with lower psychological openness and higher stigma [27]. Tan et al. reasoned that associating depression with a “weak” character could result in self-blame and shame, thus deterring help-seeking. In contrast, help-seeking propensity was linked to higher psychosocial attribution such as childhood maltreatment or trauma. Tan et al. suggested that individuals who endorsed psychosocial attributions were less likely to assume self-blame for the condition and more likely to consider the need to seek help [27].

The presentation on efficacy of medical and psychological treatments was likely to improve attitudes towards treatment as well. An additional component in our intervention that was not included in many other studies was a question-and-answer session segment with a psychiatrist and a mental health researcher. Some studies have highlighted that concerns about the characteristics of a provider was a barrier to treatment [5]. We believe that providing students with the opportunity to have their queries answered by a psychiatrist and a mental health researcher may have demystified some of the misconceived concerns, fears and scepticism regarding treatment (e.g. addictiveness of medications and side-effects, costs, confidentiality) and addressed specific concerns that were not covered by the standardised intervention. Similarly, Sharp and colleagues’ study demonstrated that providing detailed information about available mental health services positively changed student’s attitudes towards the use of such services [34].

The role of the person with lived experience must be highlighted as well. She was close in age to the target group, had sought help during the course of her education, and was experienced in public speaking and providing peer support for mental health. The sharing of her personal recovery journey and aspirations was likely to have resulted in disconfirmation of stereotypes of people with mental illness [16] and her testimonial of her recovery journey that included mental health service use may have improved perceptions towards seeking treatment. The inclusion of the Black dog video, with the male character getting better with treatment was also likely to have contributed to the positive effects on help-seeking attitudes. Both the video clip and social contact conveyed the narrative of having a meaningful life despite having a mental illness with the help of treatment. Peer story telling with recovery-oriented messages is a technique used in previous anti-stigma approaches that were associated with improvements in help-seeking intentions [35]. Emotional responses evoked during the intervention such as empathy [35] and feelings of closeness the person with lived experience [19] have been suggested to enhance intervention effects.

The relative contributions of each component of the intervention could not be determined in this study. However, it was observed that the intervention had varying effects on the components of help-seeking, with the largest effect size observed for Help-seeking Propensity both immediately and 3 months post intervention. Similarly, Gulliver’s systematic review concluded that willingness and perceived ability to seek help from a professional may be among the most amenable to change compared to other help-seeking facets [13]. This could be because Help-seeking Propensity measures beliefs about the perceived helpfulness of professional treatment which is amenable to psychoeducation and, as a construct, is relatively independent of concerns about stigma compared to the Psychological Openness and Indifference to Stigma scales. Encouragingly, help-seeking propensity was found to be the strongest predictor of intentions to engage is psychological treatment [28, 30].

Our hypothesis that the benefits of the intervention will be maintained at 3-month follow-up was not supported. While the scores for all three help-seeking subscales were significantly higher at 3-month follow-up compared to baseline, a sharp decline was observed in comparison with scores immediately after the intervention. Sharp et al’s intervention providing specific information on available mental health services yielded significant improvement in help-seeking attitudes compared to the control group that was maintained even at the 4-week follow-up [34]. With the lack of other known studies examining attitudes towards help-seeking at 3-month follow-up, our general pattern of findings nevertheless echoes past studies which saw the largest score changes in stigma-related measures at post-intervention survey point rather than at 3-month follow-up [19, 36, 37]. Transience of intervention impact has been identified as a drawback of short-term interventions such as the current one [13, 38]. It may be useful to have booster sessions in future endeavours as these sessions have been shown to improve retention and enhance intervention benefits in previous anti-stigma efforts [1, 39]. Booster sessions placed around the 3- to 4-month mark may be timely to refresh and build on the effects from the earlier intervention. On the other hand, reducing mental health stigma as a whole is arguably a formidable task, thus it has been suggested that short workshop sessions can only be part of a larger programme [40].

We observed that age and gender influenced two aspects of help-seeking, namely Psychological Openness and Help-seeking Propensity. Females were more open to acknowledging the presence of a psychological problem if they faced one and seeking help for it. This gender difference is widespread and has been found in various other studies internationally. The openness of females in acknowledging mental health problems is consistent with research suggesting that they are more likely than men to recognise and label emotional distress [41]. Men on the other hand are socialised to embody stoicism and restricted emotionality which seem in opposition to recognising emotional or psychological issues [42]. Earlier studies have also suggested that initiatives aimed at increasing men’s use of mental health services should focus on psychological openness [41].

We found an interaction between gender and pre-post Psychological Openness, with scores improving more sharply for males than females and the weakening of the intervention effects at 3 months being less pronounced than in females. This suggests that the intervention had a greater impact among males. Earlier studies have found that using role models to convey information and content that are built on positive male traits such as responsibility and strength improved help-seeking attitudes in men [43]. While we did not include a male role model, the lecture addressed differences in help-seeking between males and females and possible reasons for this which was followed with a short video narrated by a male voice of the experience of depression and how treatment improved functionality. The sharing based on lived experience of mental illness also included a description of how overcoming initial fears was important in the recovery journey.

Older age was also associated with higher Psychological Openness and Help-seeking Propensity. Several other studies have suggested that age is a predictive factor for help-seeking behaviours with younger individuals holding more negative attitudes towards mental health services [44, 45]. A study among university students found that freshmen and sophomore students were more likely to perceive barriers to mental health help-seeking than junior, senior and graduate students. This could be because younger students tend to experience more mental health issues typically associated with academic pressures as well as transition to adulthood. They may believe that their struggles are normal, wish to present themselves as self-reliant and avoid seeking help to blend in compared to their older counterparts [46]. Other studies showed that college students are exposed to and acquire more knowledge about mental illness and mental health services on campus through, for example, outreach efforts, peer-helping and psychology modules, and consequently develop more positive attitudes toward psychological help-seeking [47, 48].

Our hypothesis that those who had personal social contacts with mental illness and experience in mental health would have better attitudes towards help-seeking was partially supported. Having experience in the mental health field either through volunteer work, peer helping or taking mental health courses was associated with greater Psychological Openness and Help-seeking Propensity across all time points. In Sandhu and colleagues’ [49] study comparing implicit and explicit mental health stigma among psychiatrists, medical students and psychiatrists, only the psychiatrist group had better scores on the help-seeking/disclosure measure than the other two groups. Such findings are not surprising as these individuals are likely to believe in the value of help-seeking through their knowledge in the area. While individuals with past experience showed smaller increases in Psychological Openness immediately post-intervention, they demonstrated less decrement in scores at 3-month follow-up, supporting the earlier point that short interventions would be more effective if carried out in conjunction with other mental-health related activities (e.g. facilitated discussions on identifying and overcoming barriers to help-seeking, providing students with opportunities to gain practical experience in applying help-seeking skills learnt [1]) to improve and enhance retention of intervention benefits [39, 40].

Having friends and family with mental illness was associated with higher Psychological Openness regardless of time points. Corrigan et al. [25] and Sandhu et al. [49] found that familiarity with a person with mental illness was associated with less stigmatising attitudes. Individuals with close personal contact with a person with mental illness were less likely to view them as dangerous and demonstrated less desire for social distance. They may perceive the importance of help-seeking because they have had some first-hand knowledge of improved outcomes or understanding that symptoms of mental illness do not tend to abate without professional help. A greater rise in post-intervention but smaller fall in follow-up scores was observed among those with no family or friends with mental illness compared to those who do suggesting again that the intervention was beneficial in promoting psychological openness in a group who tended to be more reluctant to seek help at the outset.

## Conclusion

The study demonstrates the effectiveness of a brief anti-stigma intervention in improving mental health help-seeking attitudes among university students. The impact of the intervention was greatest among subgroups who had poorer help-seeking attitudes pre-intervention. This finding is important as tertiary level students were found to have among the highest treatment gap in the country for mental illnesses in an earlier local study. However, improvements in attitudes towards mental health help-seeking were observed to weaken significantly after only 3 months post-intervention. This suggests the need for continued efforts to maintain the desired effects over time.

### Strengths and limitations

Our study is the first known local study to examine the effects of an anti-stigma intervention on help-seeking attitudes among university students. Furthermore, the inclusion of the 3-month follow-up was useful in examining sustained effects of the intervention. Next, the intervention was delivered by staff from a mental health institution and was not part of course requirements, thus lending credibility to the intervention and eliminating any concern that participation will affect the students’ academic performance. Students were assured that their participation and raw data will not be shared with the university, reducing the potential for providing socially desirable responses.

There were several limitations of the study. The IASMHS has not been validated in Singapore. There was no control group to ascertain the extent to which the improved scores were due to the intervention. While, it was unlikely that factors other than the intervention could have led to improved scores immediately after the intervention, the confidence with which this result could be deduced would have been strengthened with a control group. The IASMHS assessed only attitudes towards seeking mental health services but not behaviour. The invitation to participate was sent out to a large number of students and the students who participated (*n* = 390) represented a small subset of university students. It is possible that a number of these volunteers were motivated by personal reasons, and this may have facilitated their learning and change in attitudes. Thus, the significant improvements between pre and post scores may not be generalisable to all university students. We were unable to collect data on non-response. Seventeen percent of participants were lost to follow up. Analysis were carried to compare if there were any systematic differences between those who dropped out and those who remained in the programme: We did not find any significant differences in sociodemographic variables (except ‘other’ ethnicity were more likely to drop out, although this should be interpreted with caution due to low cell count), past experience in the mental health field, having friends/family with mental illness, or in baseline IASMSHS scores. Based on this, we concluded that the 66 cases were lost to follow-up at random.

In spite of the limitations, this study presents an early attempt to examine the impact of an anti-stigma intervention among university students in Singapore. Future studies could investigate intervention characteristics that lead to improvements in help-seeking and test a further hypothesis that booster sessions at specified time-points or a combination of intervention programmes would be useful in attaining sustained outcomes.

## Data Availability

The datasets generated and/or analysed during the current study are available from the corresponding author on reasonable request.

## Abbreviations

TPB: Theory of Planned Behaviour
IASMHS: Inventory of Attitudes toward Seeking Mental Health Services
